# Supplementary material for: Yiai Fuzheng decoction inhibits triple-negative breast cancer by remodeling the immune microenvironment
Source: Front Immunol. 2025 Sep 30;16:1615631. doi: 10.3389/fimmu.2025.1615631 (PMC12518410; doi:10.3389/fimmu.2025.1615631)
Supplement: Supplementary file 2 [file DataSheet1.zip › Supplementary File 2/Treatment/C_vs_D/02_PCA/Uni_Group/C_PCA_PC12_Scoreplot.pdf]

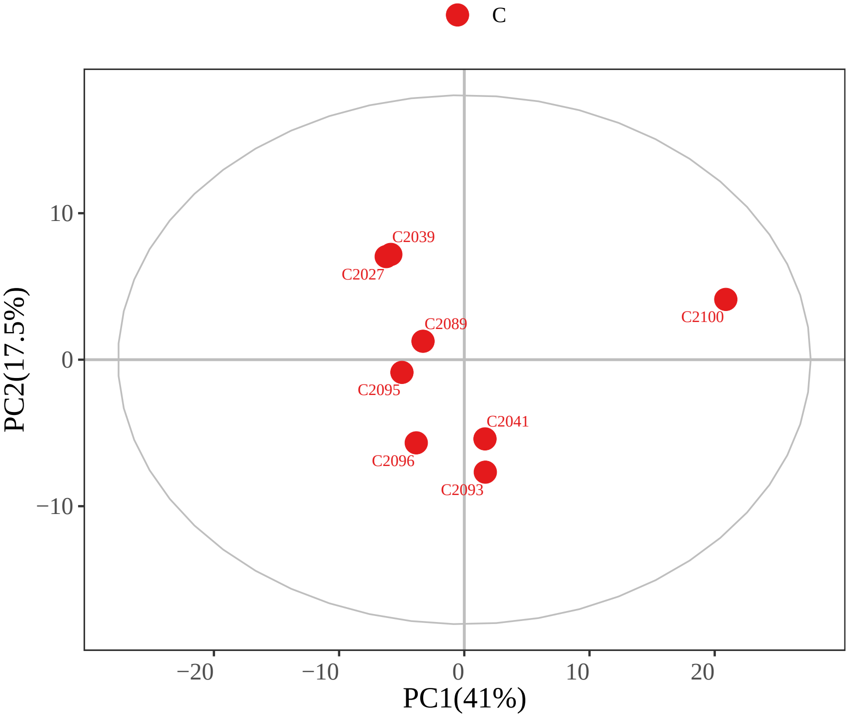

C
C2039
C2027
C2100
C2089
C2095
C2041
C2096
C2093
10
0
−10
−20
−10
0
10
20
PC1(41%)
PC2(17.5%)
